# Syndecan-1 and E-Cadherin Expression in Canine Cutaneous Squamous Cell Carcinoma

**DOI:** 10.3390/vetsci11120652

**Published:** 2024-12-14

**Authors:** Rita Files, Cláudia Cardoso, Justina Prada, Filipe Silva, Isabel Pires

**Affiliations:** 1 Department of Veterinary Sciences, University of Trás-os-Montes and Alto Douro, 5000-801 Vila Real, Portugal; ritafiles2000@gmail.com (R.F.); lauscard@gmail.com (C.C.); jprada@utad.pt (J.P.); fsilva@utad.pt (F.S.); 2 Animal and Veterinary Research Centre (CECAV), Associate Laboratory for Animal and Veterinary Sciences (AL4AnimalS), University of Trás-os-Montes and Alto Douro, 5000-801 Vila Real, Portugal

**Keywords:** cutaneous squamous cell carcinoma, syndecano-1, E-cadherin, dogs, aggressiveness

## Abstract

Cutaneous squamous cell carcinoma (CSCC) in dogs is a locally invasive tumor that typically occurs in areas of poorly pigmented skin due to sun exposure. Identifying new biomarkers, such as syndecan-1 (CD138) and E-cadherin, is fundamental for tumor diagnosis and prognosis. Dysregulation of syndecan-1, expressed in epithelial tissue, fibroblasts, and plasma cells, is associated with poor prognosis in several types of cancer. Similarly, E-cadherin, which plays a crucial role in cell adhesion and epithelial functionality, is also linked to adverse outcomes. This study evaluated the expression of syndecan-1 and E-cadherin in 47 cases of canine cutaneous squamous cell carcinoma. The results showed that the intensity of syndecan-1 decreased with increasing tumor aggressiveness, and its presence in the stroma was significantly associated with tumor grade. E-cadherin also demonstrated a decrease in intensity with increasing malignancy. However, the association between syndecan-1 and E-cadherin was not statistically significant. E-cadherin reduction and stromal syndecan-1 positivity seem to be associated with tumor aggressiveness in canine cutaneous squamous cell carcinoma. Further studies are needed to explore their roles in tumor progression.

## Simple Summary

This study focuses on cutaneous squamous cell carcinoma, a common type of skin cancer in dogs that usually appears in areas of the skin with little pigment, often due to sun exposure. The research aimed to explore two specific markers, syndecan-1 and E-cadherin, which can help scientists understand how aggressive the tumor might become. We examined 47 cases of this cancer and found that as the tumors became more aggressive, the levels of these markers decreased. Syndecan-1 was found more in the tissue surrounding the tumors, and its presence was linked to how severe the cancer was. E-cadherin also showed lower levels in more severe cases. These findings could provide veterinarians with valuable information, potentially aiding in predicting tumor progression. More studies are needed to understand how these markers affect cancer growth fully.

## 1. Introduction

Cutaneous squamous cell carcinoma (CSCC) is a type of cancer originating in the stratified epithelium of the skin, arising from the abnormal proliferation of keratinocytes [1,2,3]. Known for having the highest mutational burden among solid tumors, CSCC is commonly found in areas exposed to sunlight, such as the head and neck. Several risk factors contribute to its development in humans, including exposure to ultraviolet (UV) radiation, being male, advanced age, fair skin, immunosuppression, smoking, human papillomavirus (HPV) infection, skin ulcers, and chronic conditions like leukemia and non-Hodgkin’s lymphoma [4,5,6,7,8].

In animals, particularly dogs, CSCC is also a common type of skin cancer, accounting for about 5.4% of all cutaneous tumors. When CSCC occurs in the skin, it exhibits locally aggressive behavior, with a higher likelihood of metastasis when located on a digit. In most cases, the tumor remains locally invasive, with lower chances of spreading to other body parts [4,5,9]. CSCC is often seen in areas of the skin that are either minimally pigmented or depigmented, particularly in sun-exposed regions [10,11,12]. Certain dog breeds, such as Beagles, Pit Bulls, Schnauzers, Basset Hounds, Collies, and Dalmatians, are more prone to developing CSCC due to their greater likelihood of having depigmented skin [10,11,12]. When cases of CSCC in dogs are at an advanced stage, they tend to have a poor response to treatment [13,14].

The mutations that lead to the development of CSCC are often the result of endogenous factors such as DNA repair errors, mitotic errors, reactive oxygen species, and genome editing [15,16,17,18]. For the mutations to proceed and give rise to the tumor, they occur mainly in long-lived cells within the epidermis, in the basal layer area [15,16,17,18].

The CD138 gene encodes syndecan-1, a transmembrane proteoglycan in the syndecan family, which includes SDC2 (fibroglycan), SDC3 (N-syndecan), and SDC4 (amphoglycan or ryudocan) [19,20,21,22]. Syndecan-1 is primarily expressed in epithelial and plasma cells, playing a key role in regulating cell adhesion to the extracellular matrix and influencing cell migration and proliferation. Structurally, syndecan-1 contains extracellular, transmembrane, and cytoplasmic domains, which are linked to glycosaminoglycan (GAG) chains, primarily heparan sulfate (HS) and chondroitin sulfate (CS) [21,23,24]. These GAG chains facilitate interactions with various molecules critical for cell signaling and maintaining cell morphology [23,25,26,27]. Through their HS chains, they can bind growth factors and mediate signaling pathways, such as VEGF, WNT, and HGF, as co-receptors. Syndecan-1 can be cleaved from the cell surface by matrix metalloproteinases (MMP9), becoming soluble and capable of binding to other molecules. Loss of syndecan-1 expression in tumor cells leads to reduced cell–cell cohesion, increasing the potential for tumor invasion and metastasis. Conversely, syndecan-1 expression in the tumor stroma can enhance tumor angiogenesis by binding to growth factors like basic fibroblast growth factor [19,20,26,28].

Cadherins are a large family of cell surface proteins involved in calcium-dependent cell–cell adhesion, essential for normal development. They are classified into classical and non-classical types, with classical cadherins further divided into type 1 and type 2. Type 1 cadherins, such as E-cadherin (epithelial-cadherin) and N-cadherin (neuronal-cadherin), are found in tissues that require strong cell adhesion [29,30,31]. In contrast, type 2 cadherins, like cadherin 7 and 11, are expressed in highly motile cells. E-cadherin, encoded by the CDH1 gene, is crucial for embryonic development and maintaining epithelial integrity. It plays a significant role in forming stable adherens junctions, influencing cell polarity, maintaining tissue cohesion, and preventing invasive cellular behavior [32,33,34]. E-cadherin emerges as a pivotal molecule whose dysregulation holds significant implications, particularly in the context of cancer. While it has been extensively studied in various cancer types, its exact role remains controversial [35,36]. Traditionally considered a tumor suppressor, recent research reveals a more nuanced understanding, especially in advanced cancer stages where it can paradoxically stimulate cell migration, invasion, and tumor progression [35,36].

In human squamous cell carcinoma, syndecan-1 and E-cadherin have shown promise as markers. However, no studies have been published regarding their relationship with animal tumor aggressiveness. Syndecan-1 and E-cadherin are cell adhesion molecules expressed mainly on the surface of adult epithelial cells [37,38,39]. They appear to be co-regulated and may act together to stabilize the epithelium. E-cadherin and syndecan-1 expression loss is observed in malignant transformation and invasion and is associated with epithelial–mesenchymal transition (EMT) [40,41,42,43].

Therefore, this study aims to evaluate the immunohistochemical expression of SDC1 and E-cadherin in canine cutaneous squamous cell carcinoma and to explore the relationship between these markers and the histological grade of malignancy.

## 2. Materials and Methods

### 2.1. Clinical and Histopathological Evaluation

This study analyzed 47 cases of cutaneous squamous cell carcinomas in dogs. The tumor samples were obtained from the Histology and Pathology Laboratory of the University of Trás-os-Montes and the Alto Douro archive. These tumors were collected during surgical procedures or necropsies, previously fixed in 10% buffered formalin, and embedded in paraffin. Clinical data, such as age, gender, and breed, were recorded for each animal.

Most of the tumors occurred in purebred animals, particularly in Boxer dogs (n = 4), Basset Hound (n = 2), Bobtail(n = 1), Bulldog Castro Laboreiro Dog (n = 1), Cocker Spaniel (n = 1), Dalmatian dog (n = 1), German Sheperd (n = 1), Siberian Husky (n = 1), Labrador Retriever (n = 2), Pincher (n = 2), Pit Bull (n = 2), Pointer (n = 2), Poodle (n = 2), Rottweiler (n = 1), Spanish Hound (n = 1), Giant Schnauzer (n = 1), Estrela mountain dog (n = 1), Irish Setter (n = 1), Sharpei (n = 1), Westhighland Terrier (n = 1), and Yorkshire (n = 2). In 15 cases, the animals were of mixed breeds. Regarding sex distribution, 24 (51%) were females, and 23 (49%) were males. The animals’ ages ranged from 1 month to 17 years, with an average age of 9.2 years (±4.16).

Two pathologists (IP and JP) conducted independent analyses for each sample, examining all the slides in each section in detail. The histopathological diagnosis followed the World Health Organization (WHO) classification of animal tumors [44]. A Nikon Eclipse E600 microscope with a Nikon DXM1200 digital camera (Nikon Instruments Inc., Melville, NY, USA) was used for microscopic observations and image capture.

The evaluated parameters included the degree of keratinization, nuclear pleomorphism, mitotic activity, and the presence of emboli. Keratinization levels were categorized based on the percentage of tumor cells showing keratinization, resulting in the following groups: I (>50% keratinized cells), II (20–50% keratinized cells), and III (0–20% keratinized cells). Nuclear pleomorphism was classified into three levels: I (minimal, with >75% of cells appearing mature), II (moderate, with 50–75% mature cells), and III (severe pleomorphism, <50% mature cells). Mitotic count was determined over ten high-power fields (HPF) and divided into categories: I (0 to 1 mitosis/HPF), II (2 to 3 mitoses/HPF), and III (≥4 mitoses/HPF) [44].

The histological degree of malignancy was determined, as described by [45], into three categories (Grade I, II, III) described in Table 1.

### 2.2. Immunohistochemical Evaluation for SDC1 and E-Cadherin

For immunohistochemical analysis, 3-µm-thick sections were mounted on silane-coated slides [46], using the NovolinkTM Polymer Detection System (Leica Biosystems^®^, Newcastle, UK), with 3,3′-diaminobenzidine tetrachloride (DAB) as the chromogen, according to the manufacturer’s instructions.

The slides were dehydrated using decreasing concentrations of ethanol (100%, 95%, 80%, and 70%) for 5 min in each solution. After this process, they were washed in running water for 10 min. Antigenic recoating was carried out with citrate buffer (10 mM, pH 6.0 ± 0.2) in a 20-min cycle in a microwave at 750 W. The slides were then incubated with Peroxidase Block from the Novolink Novocastra^®^ Kit polymer detection system for 5 min and subsequently incubated with Protein Block^®^ for 5 min. The slides were incubated with the Anti-CD138 primary antibody (Abcam, Cambridge, UK) for 24 h at a dilution of 1:50. The following day, after washing the slides in PBS, they were incubated with the secondary antibody (Post Primary Antibody^®^) (Leica Biosystems ^®^, Newcastle, UK) for 30 min, followed by 30 min with Nova Link polymer^®^. The slides were then incubated with the DAB^®^ chromogen (Novo-Castra) (1:20 dilution) for 10 to 12 min. After this period, the slides were placed in running water for 5 min. Counter-staining was carried out with Gill’s hematoxylin for 1 min, followed by 10 min in running water. The slides were then dehydrated and diaphanized in xylene for 15 to 45 min. Finally, the slides were mounted with Entellan^®^. The same procedure was carried out for the anti-E-cadherin antibody (Zymed Laboratory, San Francisco, CA, USA), diluted at 1:50. Both antibodies were used in canine samples in previous works [43,47]. The specificity of the staining was confirmed using negative and positive controls (plasma cells as internal controls and canine samples with lymphoplasmocytic disease for CD138 and for E-Cadherin, samples of dog skin and cutaneous papilloma).

### 2.3. Quantification of the Immunoreactivity

All samples were independently evaluated by two observers (IP and JP) blinded to the clinical and pathological characteristics of the cases. To assess interobserver agreement, the kappa statistic was calculated for all scoring categories, showing a range of substantial to almost perfect agreement (κ = 0.68–0.85). In instances of discrepancies in scoring, a third reviewer (RF) was consulted. A consensus discussion was subsequently held to determine the final score.

#### 2.3.1. Syndecan-1

Syndecan-1 expression in tumor cells was assessed semi-quantitatively based on the intensity adapted from [48]. Immunoreactivity was considered when a brownish marking was observed on the tumor cells’ membrane and/or cytoplasm [48,49]. The intensity was evaluated as: negative (0), weak (1), moderate (2), and strong (3) [48]. The scoring was based on the staining intensity observed in the tumor cells relative to adjacent normal epidermis and in samples of normal canine skin, which is considered strong (3).

In addition, the presence of this marker in the stroma cells was examined, and it was considered positive when detected in at least 5% of the cells, regardless of intensity [50].

#### 2.3.2. E-Cadherin

E-cadherin immunoreactivity was assessed semi-quantitatively in terms of the intensity of membrane reaction, as described previously. The intensity of expression was classified as follows: negative (0), weak (1), moderate (2) and strong (3) [51,52]. Staining intensity was assessed relative to adjacent normal epidermis or control samples from normal canine skin. Staining was considered strong (3) when its intensity was comparable to that of the normal epidermis.

### 2.4. Statistical Analysis

SPSS (version 21; SPSS Inc, Chicago, Illinois, USA) was used to perform all statistical tests. Clinicopathological characteristics were presented as absolute and relative frequencies, and a descriptive analysis was conducted. The chi-squared (χ2) test and Fisher’s exact test were employed to evaluate the association between biomarkers and histopathological characteristics, with descriptive analysis also performed. Given the small sample size and the potential for some expected frequencies to fall below 5, a continuity correction was applied to the chi-square test. Cramer’s V and φ coefficient tests were used to evaluate the association’s strength. Effect sizes were interpreted as follows: <0.10: negligible association; 0.10–0.30: small association; 0.30–0.50: moderate association; V ≥ 0.50: strong association [53].

A logistic regression model was constructed to quantify the relationships between immunohistochemical markers (e.g., syndecan-1 and E-cadherin expression) and histological parameters. Syndecan-1 expression (intensity and stromal positivity) and E-cadherin intensity were included as independent variables, with histological grade as the primary dependent variable. Additional models were developed to assess associations with other histological parameters. To ensure the robustness of the regression model, the following checks were conducted: multicollinearity, model adequacy, independence of errors, and residual analysis. The Variance Inflation Factor (VIF) was calculated for all independent variables to assess multicollinearity. VIF values below 10 confirmed the absence of significant collinearity among the predictors. The overall adequacy of the multinomial logistic regression model was evaluated using log-likelihood, AIC, and BIC. Regarding the independence of errors, the study’s design ensured independence among observations. Deviance and Pearson residuals were examined to identify potential patterns or outliers. No significant deviations or problematic trends were observed. Odds ratios (ORs), confidence intervals (CIs), and *p*-values were calculated to quantify the strength and direction of associations. A *p*-value < 0.05 was considered statistically significant.

## 3. Results

### 3.1. Histopathological Evaluation

A total of 47 tumors diagnosed as squamous cell carcinoma of the skin were studied. Twelve cases (26%) showed more than 50% keratinized cells (I), 21 cases (45%) had 20–50% keratinized cells (II), and 14 cases (29%) had 0–20% keratinized cells (III).

Nuclear pleomorphism was mild in 9 cases (19%), corresponding to Grade I, moderate in 20 cases (43%), classified as Grade II, and severe in 18 cases (38%). The distribution of mitotic count was as follows: 17 cases (36%) were categorized as Grade I, 17 cases (36%) as Grade II, and 13 cases (28%) as Grade III. Emboli were present in 15 cases (32%).

**Figure 1:**
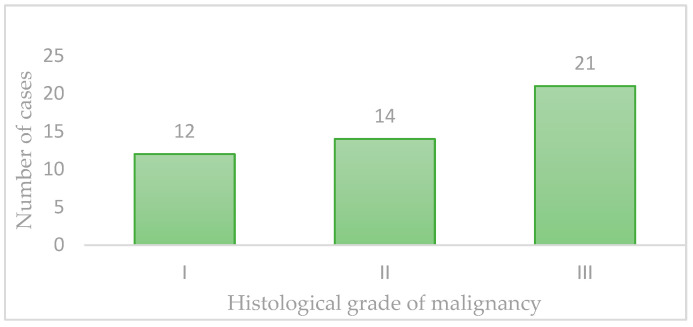
Histological grade of malignancy.

Regarding malignancy grading, out of the 47 cases analyzed, 12 cases (25.5%) were classified as Grade I, 14 cases (29.8%) as Grade II, and 21 cases (44.7%) as Grade III (Figure 1).

### 3.2. Immunohistochemical Evaluation

#### 3.2.1. Syndecan-1 Immunoexpression

Syndecan-1 staining was primarily observed in the cytoplasm and membrane of tumor cells, with occasional nuclear localization. The staining pattern varied across the samples, showing differences in intensity, particularly in invasive areas where it was less intense. Of the 47 cases, 30 (64%) were positive for syndecan-1 expression in tumor cells, while 17 (36%) were negative. Among the positive cases, 11 (23%) exhibited weak intensity (I), 16 (34%) had moderate intensity (2), and only 3 cases (6%) showed strong intensity.

Except for mitotic count, no significant associations were observed when comparing syndecan-1 expression in tumor cells with histopathological characteristics. A statistically significant association was found between syndecan-1 labeling intensity and mitotic count (*p* = 0.028; Cramer’s V = 0.39, moderate association), Table 2. Among the cases with a high mitotic count, 7 out of 13 (53.8%) showed negative syndecan-1 expression, and 5 out of 13 (38.5%) had intensity 1, suggesting that as the number of mitoses increases, syndecan-1 expression tends to decrease.

**Figure 2:**
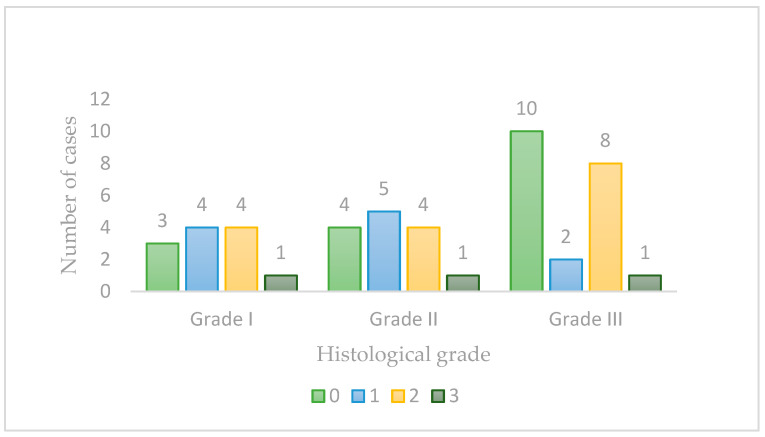
Syndecan-1 labeling intensity in tumors with different histological grades of malignancy.

Regarding histological grade, there were no statistically significant differences (*p* = 0.552; Cramer’s V = 0.23, weak association). However, a decreased labeling intensity was observed in less differentiated tumors, with 47.6% of Grade III cases showing negative syndecan-1 expression (10 out of 21 cases), Figure 2. For other histopathological parameters, no statistically significant associations were observed: nuclear pleomorphism (*p* = 0.48, Cramer’s V = 0.24, weak association), emboli presence (*p* = 0.55, Cramer’s V = 0.23, weak association), keratinization (*p* = 0.98, Cramer’s V = 0.11, negligible association), Table 2.

**Figure 3:**
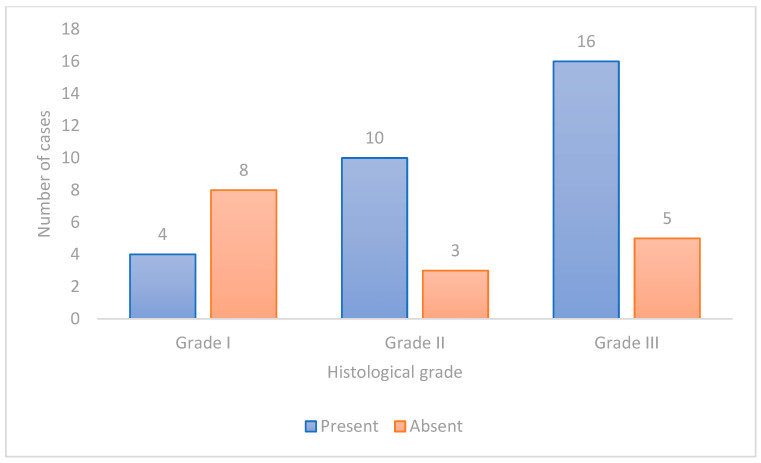
Syndecan-1 stromal immunoexpression in tumors with different histological grades of malignancy.

Stromal positivity for syndecan-1 was observed in 30 cases (65.2%). A statistically significant association was found between stromal positivity and keratinization/differentiation (*p* = 0.026; Cramer’s V = 0.40; moderate association) as well as histological grade (*p* = 0.026; Cramer’s V = 0.40, moderate association), Figure 3. Among the 30 positive cases, 16 (53.3%) were classified as Grade III, 10 (33.3%) as Grade II, and only 4 (13.3%) as Grade I, suggesting that less differentiated tumors and those with higher grades of malignancy generally exhibited stromal positivity.

For other histopathological parameters, no statistically significant associations were observed: mitotic count (*p* = 0.058; Cramer’s V = 0.35, moderate association), nuclear pleomorphism (*p* = 0.070; Cramer’s V = 0.34, moderate association) and emboli presence (*p* = 0.48, φ coefficient = 0.216, weak association).

**Figure 4:**
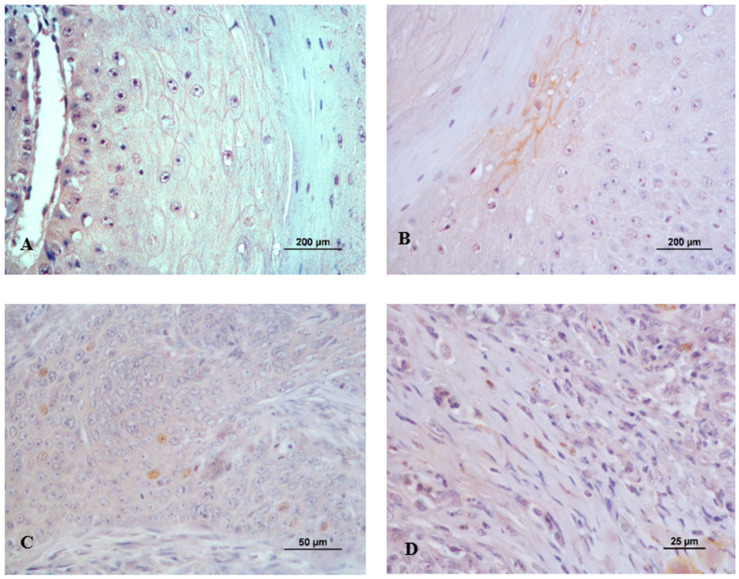
Syndecan-1 immunoexpression in canine cutaneous squamous cell carcinoma. (**A**) Membranous labeling with moderate intensity (2) in a well-differentiated tumor (Grade I); (**B**) Membranous and cytoplasmatic expression with moderate intensity (2) in a well-differentiated tumor (Grade I); (**C**) Week cytoplasmatic and nuclear labeling in moderately differentiated tumor (Grade II); (**D**) Stroma immunoexpression in a poorly differentiated tumor (Grade III).

Figure 4A–D shows the immunoexpression of syndecan-1 in both tumor and stromal cells.

#### 3.2.2. E-Cadherin Immunoexpression

E-cadherin expression was assessed based on the intensity of membranous labeling. Among the 47 cases, 11 showed weak expression (Grade I), 23 showed moderate expression (Grade II), and 13 exhibited strong expression (Grade III) for E-cadherin.

Considering the histopathological parameters, there was a statistically significant association between the intensity of E-cadherin staining and keratinization/differentiation (*p* = 0.035; Cramer’s V = 0.422, moderate association) and nuclear pleomorphism (*p* = 0.041; Cramer’s V = 0.398, moderate association). Tumors with lower differentiation and higher pleomorphism generally displayed weaker E-cadherin intensity, Table 3.

**Figure 5:**
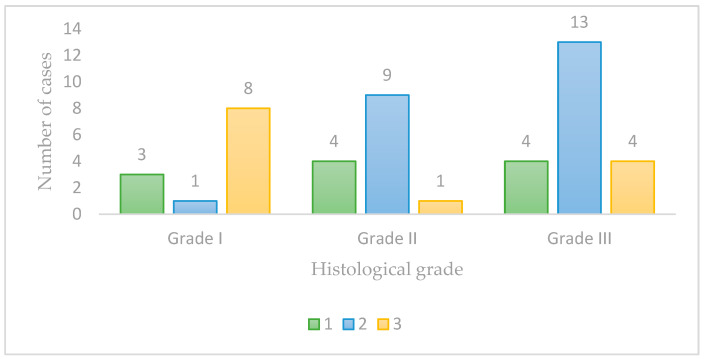
E-cadherin labeling intensity in tumors with different histological grades of malignancy.

The histological grade of malignancy was also significantly associated with the intensity of E-cadherin staining (*p* = 0.005; Cramer’s V = 0.40, moderate association), with Grade I tumors showing higher intensity compared to Grade II and III tumors (Figure 5), Table 3.

For other parameters: mitotic count (*p* = 0.32, Cramer’s V = 0.22, weak association) and emboli presence (*p* = 0.48, Cramer’s V = 0.24, weak association), no statistically significant associations were observed (Table 3).

**Figure 6:**
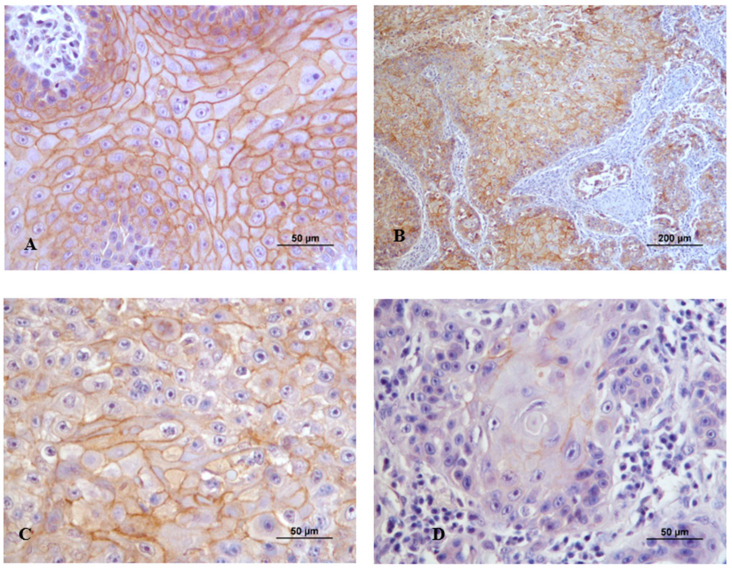
E-cadherin immunoexpression in canine cutaneous squamous cell carcinoma. (**A**) Membranous labeling with strong intensity (3) in a well-differentiated tumor (Grade I); (**B**) Membranous and cytoplasmatic expression with strong intensity (3) in a moderately differentiated tumor (Grade II); (**C**) Moderate membranous labeling in poorly differentiated tumor (Grade III); (**D**) Weak membranous labeling in poorly differentiated tumor (Grade III).

Figure 6A–D shows the immunoexpression of E-cadherin in tumor cells.

### 3.3. Regression Analysis

The regression model findings identified syndecan-1 stromal positivity and E-cadherin intensity as significant predictors of tumor aggressiveness. Syndecan-1 stromal positivity was significantly associated with higher histological grades (OR = 3.12, 95% CI: 1.52–6.39, *p* = 0.002). E-cadherin intensity showed an inverse association with histological grade, with weaker intensity associated with higher grades (OR = 0.45, 95% CI: 0.25–0.78, *p* = 0.004). For nuclear pleomorphism, E-cadherin intensity demonstrated a similar trend (OR = 0.49, 95% CI: 0.28–0.87, *p* = 0.009).

### 3.4. Association Between Syndecan-1 and E-Cadherin

Regarding the possible association between both markers, no association was found between the expression of the two markers (*p* = 0.898), i.e., a decrease in one marker was not correlated with a reduction in the other.

## 4. Discussion

Squamous cell carcinoma (SCC) results from a complex process involving the accumulation of various genetic, epigenetic, and environmental changes. These alterations can activate oncogenes or inactivate tumor suppressor genes, activating and deactivating different cellular pathways [13,54,55].

This study analyzed 47 canine cutaneous squamous cell carcinomas, with an average age of 9.2 years. Although there is no consensus in the literature on the specific age range for this type of cancer, it is estimated to occur between 6 and 13 years, with a higher prevalence in older dogs [56,57].

The genetic variation among dogs is closely tied to breed history, influencing physical and behavioral traits and predisposition to age-related diseases. This genetic diversity between breeds and homogeneity within each breed helps identify hereditary factors linked to specific diseases. For instance, Bernese Mountain Dogs are ten times more likely to die from cancer compared to other breeds, even after adjusting for age and sex [58]. Moreover, dogs with depigmented skin, light-colored coats, and medium to large body sizes are more prone to developing squamous cell carcinoma. Giant Schnauzers, Gordon Setters, Briards, Kerry Blue Terriers, and Standard Poodles are among the most susceptible [59,60,61]. In our results, 15 of the 47 dogs were of mixed breeds and the remaining of diverse purebred animals: Boxers, with four cases, Basset Hound (n = 2), Bobtail (n = 1), Bulldog Castro Laboreiro Dog (n = 1), Cocker Spaniel (n = 1), Dalmatian dog (n = 1), German Sheperd (n = 1), Siberian Husky (n = 1), Labrador Retriever (n = 2), Pincher (n = 2), Pit Bull (n = 2), Pointer (n = 2), Poodle (n = 2), Rottweiler (n = 1), Spanish Hound (n = 1), Giant Schnauzer (n = 1), Estrela mountain dog (n = 1), Irish Setter (n = 1), Sharpei (n = 1), Westhighland Terrier (n = 1), and Yorkshire (n = 2). Thirty-two of the cases were purebred dogs.

There is no evidence in the literature suggesting a link between sex and this specific tumor [10,57]. Our findings indicated that 51% of the cases were female and 49% were male, showing no significant difference. Other studies have reported a higher incidence in males than females [62].

The expression of syndecan-1 and E-cadherin was studied in 47 canine squamous cell carcinoma using immunohistochemistry. This method is commonly applied in pathology laboratories due to its significance in diagnosis, prognosis, and treatment [63,64].

Syndecan-1 is generally expressed in epithelial, plasma, mesenchymal, and fibroblast cells [50]. It plays crucial roles in cell–cell interaction, adhesion, migration, angiogenesis, and proliferation [65,66,67]. This molecule is essential in maintaining epithelial morphology and inhibiting invasion, and it is closely associated with aggressiveness in various cancers, which can either increase or decrease depending on the cancer type [42,68].

When analyzing the intensity of syndecan-1 expression, although no statistically significant association was identified between the intensity of this marker and histological grade, our results indicate a greater number of Grade III cases with weak or absent staining. This suggests a possible decrease in syndecan-1 expression as tumor aggressiveness increases. Several studies have documented syndecan-1 (SDC1) expression changes across various carcinomas. In prostate cancer, the transcription factor Zeb1 binds to the E-box in the SDC1 promoter, suppressing its expression. Similarly, in gynecological cancers, estrogen receptor α signaling downregulates SDC1 expression. Research on the SDC1 promoter in murine models has identified key regulatory elements, including the TATA box, CAAT box, E-box, and binding sites for transcription factors Sp1 and NF-kB. These findings highlight diverse mechanisms underlying altered SDC1 expression in different cancer types [19,69,70].

These findings are consistent with those reported in other studies on dogs, including invasive and metastatic oral melanoma [71], oral squamous cell carcinoma [43], as well as mammary carcinomas [49]. Some studies on human squamous cell carcinoma (SCC) have reported similar findings. This includes observations in oral squamous cell carcinoma [72], lung SCC, and head and neck cancers [48,73,74,75], as well as renal cell carcinoma [76] and non-small cell lung carcinoma [41,77]. We also observed a statistically significant relationship with the number of mitoses, suggesting that increased proliferation of tumor cells is associated with decreased syndecan-1 expression. Interestingly, only three cases showed strong staining, suggesting that the reduced expression of syndecan-1 could be an early event in the neoplastic transformation of SCC in dogs.

One of the primary questions from analyzing these results is: “How does the loss of syndecan-1 expression occur?” The literature indicates that this loss can happen through two main mechanisms: negative transcriptional regulation and cleavage of the ectodomain of syndecan-1 induced by constitutive or extracellular matrix metalloproteinases (MMPs) [78]. The loss of syndecan-1 from the cell membrane decreases epithelial cell adhesion, reducing binding to the extracellular matrix and facilitating tumor cell migration and invasion. Numerous studies highlight that the reduction of this molecule is a significant marker in the epithelial–mesenchymal transition (EMT) process [41].

In our study, a statistically significant relationship between histological grade and the presence of syndecan-1 in the stroma was found, suggesting that tumors with greater malignancy express this marker in the stroma, as described in other human tumor studies [21,79]. There are no studies in dogs that have obtained similar results. However, stromal syndecan-1 expression has also been observed in human squamous cell carcinoma cases [80]. The tumor stroma plays a fundamental role in cancer progression, regulating processes such as cell proliferation, invasion and metastasis. The extracellular matrix (ECM) remodeled in the stroma comprises proteoglycans (PGs), collagens, fibronectin, laminins, hyaluronic acid, growth factors and stromal cells. This composition creates a microenvironment that favors tumor aggressiveness. Proteoglycans, such as syndecans, are essential components of the tumor microenvironment, playing crucial roles in cell adhesion, proliferation and motility. Changes in the expression of these components, such as syndecan-1, directly impact cancer progression, making them valuable biomarkers and potential therapeutic targets [19]. Our findings could suggest that a decreased expression of syndecan-1 on the cell membrane and its transition to the stroma could be associated with epithelial tumor progression, as described in human tumors [81].

The other molecule studied, E-cadherin, is known for mediating cell–cell and cell–matrix adhesion, playing a crucial role in establishing cellular polarity and differentiation [82]. Since most tumors, in both humans and dogs, have an epithelial origin, E-cadherin has been extensively investigated for its role in tumorigenesis [83]. Its function is generally regarded as tumor-suppressive, as it helps maintain cells in their normal state [83]. In this study, an association was observed between the intensity of E-cadherin expression and histopathological features such as keratinization/differentiation and nuclear pleomorphism. These results indicate that as cells become less differentiated, there is a corresponding decrease in E-cadherin expression, linking its loss to changes in cellular morphology and the emergence of undifferentiated and atypical cells. This behavior is consistent with findings in canine oral squamous cell carcinoma [84], canine cutaneous squamous cell carcinoma [85], and canine mammary carcinoma [86,87].

Similarly, studies in human cancer have reported comparable patterns in oral squamous cell carcinoma [43,88,89], head and neck carcinoma [90], and cutaneous squamous cell carcinoma [91]. Moreover, a significant association was found between the intensity of E-cadherin expression and the histological grade of malignancy, suggesting that as malignancy increases, E-cadherin expression diminishes. The loss of E-cadherin is recognized as a marker for the diagnosis and prognosis of epithelial cancers [92]. It has been reported that decreased expression is associated with larger tumor size, high histological grade, invasion, metastasis, and increased mitotic index, which is consistent with our findings [92]. The CDH1 gene, known to act as a tumor suppressor, plays an essential role in maintaining cell–cell adhesion, ensuring the orderly arrangement and stratification of epithelial cells. In addition, they also describe that the in vitro studies indicate that loss of CDH1 expression or function can trigger the activation of transcription factors linked to epithelial–mesenchymal transition (EMT), a process that contributes significantly to the occurrence of metastasis in cancer cells. It has been described that hypermethylation of the CDH1 promoter is the predominant mechanism of E-cad loss in several types of cancer, including breast cancer [36,93,94,95].

In many cancers, the loss of E-cadherin is attributed to transcription factors such as SNAIL, SLUG, TWIST, ZEB1, and ZEB2, which bind to E-box sequences in the promoter region of the CDH1 gene and negatively regulate its expression, often through promoter methylation [96].

Domestic dogs are considered relevant models for cancer study due to their shared physical and chemical environments with humans, as well as the presence of approximately 650 Mb of ancestral genetic sequence. Several types of cancer, including squamous cell carcinoma, show remarkable similarities between the two species [60,64,97,98,99]. Studies reveal shared biological traits, such as telomere and telomerase activity, molecular equivalences, similar histological subtypes, and comparable expression patterns of markers identified via immunohistochemistry in various tumors. Additionally, canine SCC, arising spontaneously, better reflects human tumors’ biological heterogeneity and complexity compared to traditional experimental models, further underscoring their relevance [60,64,97,98,99].

While these similarities highlight the value of studying naturally occurring cancers in dogs as models for understanding, diagnosing, and developing new therapeutic strategies for human cancers, it is important to acknowledge the limitations in directly translating findings between species. Differences in tumor biology, immune responses, and treatment outcomes must be carefully considered to avoid overgeneralization [60,64,97,98,99].

This preliminary study highlights limitations such as a small sample size, breed variability, and the subjective nature of semi-quantitative scoring methods, which may impact result reproducibility. Additionally, the lack of standardized thresholds for syndecan-1 expression and the absence of molecular data on gene expression or prognostic correlations pose significant challenges. Future studies should address these limitations by incorporating larger, more homogeneous samples, automated analysis methods, and genetic evaluations. Despite these constraints, our results provide initial insights into the expression of syndecan-1 and E-cadherin in canine cutaneous tumors, laying the groundwork for further investigations into their roles in tumor progression and prognosis.

Syndecan-1 stromal positivity is significantly associated with higher histological grades in canine cutaneous squamous cell carcinoma, making it a potential diagnostic tool for tumor aggressiveness and prognostic marker. Similarly, E-cadherin intensity inversely correlates with histological grade and nuclear pleomorphism, suggesting that weaker E-cadherin expression could be a marker for less differentiated, more aggressive tumors. Additionally, targeting the pathways involved in syndecan-1 and E-cadherin expression could offer new therapeutic opportunities [100]. In the case of syndecan-1, there is evidence that chemotherapy can induce the release of its ectodomain, which can have a negative impact on the response to treatment [66,101]. Additionally, promising therapies to reduce its elimination to limit tumor progression include heparanase and MMP inhibition, synthetic proteoglycans mimicking SDC1, and strategies targeting growth factor interactions [26,28,102]. Therapeutic strategies targeting E-cadherin have shown promise in clinical and preclinical models for suppressing metastasis and restoring epithelial characteristics, though studies in dogs remain limited. These approaches include gene therapy (e.g., miR-200 and siRNA delivery via viral or non-viral vectors), epigenetic interventions (demethylating agents and HDAC inhibitors), and pharmacological treatments (e.g., aspirin, NSAIDs, adriamycin, and regorafenib) [36,103,104].

Access to detailed information on the role of these biomarkers makes it possible to personalize therapeutic approaches according to the specific characteristics of each case. Thus, both syndecan-1 and E-cadherin are potentially valuable tools in clinical practice, contributing to the prediction of tumor behavior and the choice of interventions that improve clinical outcomes [105].

## 5. Conclusions

These results demonstrate that the decrease of E-cadherin expression and the transition of syndecan-1 expression to the stroma are significantly associated with histological grade, suggesting their potential involvement in tumor progression. However, as this study demonstrates associations rather than causality, further comparative and mechanistic studies are necessary to understand the biological and clinical significance of these observations.

## Figures and Tables

**Table 1:** Classification of histological grade, based on [45].

Histological Grade	Histological Features
Grade I (Well differentiated)	High cytoplasm/nucleus ratio (nuclear pleomorphism-1) Vitreous atypia Prominent keratinization (1)
Grade II (Moderately differentiated)	Some keratinization (2) No spindled or anaplastic cells (nuclear pleomorphism-1 or -2)
Grade III (Poorly differentiated)	Sparse to absent keratinization (3) Spindled or anaplastic cells (nuclear pleomorphism-3) Difficult to identify as SCC

**Table 2:** Association between histological characteristics and tumor cells and stroma syndecan-1 immunoexpression.

Histological Parameter	Tumor Cells Syndecan-1 Immunoexpression				Stroma Syndecan-1 Immunoexpression	
	0	1	2	3	Negative	Positive
Degree of keratinization						
1	4 (33.3%)	3 (25.0%)	4 (33.3%)	1 (8.3%)	8 (66.7%)	4 (33.3%)
2	7 (33.3%)	6 (28.6%)	7 (33.3%)	1 (4.8%)	5 (25%)	15 (75%)
3	6 (42.8%)	2 (14.3%)	5 (35.7%)	1 (7.1%)	3 (21.3%)	11 (78.6%)
	*p* = 0.98				*p* = 0.026	
Nuclear pleomorphism						
1	1 (11.1%)	4 (44.5%)	3 (33.3%)	1 (11.1%)	6 (66.7%)	3 (33.3%)
2	8 (40.0%)	5 (25.0%)	6 (30%)	1 (5%)	5 (26.3%)	14 (73.7%)
3	8 (40.0%)	2 (11.1%)	7 (38.9%)	1 (5.6%)	5 (27.8%)	13 (72.2%)
	*p* = 0.48				*p* = 0.07	
Mitotic count						
1	4 (23.5%)	6 (35.3%)	6 (35.3%)	1 (5.9%)	8 (47.0%)	9 (52.9%)
2	6 (35.3%)	0	9 (52.9%)	2 (11.8%)	7 (43.7%)	9 (56.2%)
3	7 (53.8%)	5 (38.8%)	1 (7.7%)	0	1 (7.7%)	12 (92.3%)
	*p* = 0.028				*p* = 0.058	
Emboli						
Present	7 (46.7%)	2 (13.3%)	6 (40.0%)	0	3 (20%)	12 (80%)
Absent	10 (31.3%)	9 (28.1%)	10 (31.2%)	3 (9.4%)	13 (41.9%)	18 (38.3%)
	*p* = 0.55				*p* = 0.48	
Histological Grade						
I	3 (17.6%)	4 (23.5%)	4 (23.5%)	1 (5.9%)	8 (66.7%)	4 (33.3%)
II	4 (28.6%)	5 (35.7%)	4 (28.6%)	1 (7.1%)	3 (23.1%)	10 (76.9%)
III	10 (47.6%)	2 (9.5%)	8 (38.1%)	1 (4.8%)	5 (23.8%)	16 (76.2%)
	*p* = 0.552				*p* = 0.026	

**Table 3:** Association between histological characteristics and the intensity of E-cadherin.

Histological Parameter	E-Cadherin Immunoexpression		
	1	2	3
Degree of keratinization			
1	3 (25%)	2 (16.7%)	7 (58.3%)
2	6 (28.6%)	11 (52.4%)	4 (19.1%)
3	2 (14.3%)	10 (71.4%)	2 (14.3%)
	*p* = 0.035		
Nuclear pleomorphism			
1	1 (11.1%)	2 (11.1%)	6 (66.7%)
2	6 (30%)	12 (60%)	2 (10%)
3	4 (22.2%)	9 (50%)	5 (27.8%)
	*p* = 0.0422		
Mitotic count			
1	4 (23.5%)	6 (35.3%)	7 (41.2%)
2	3 (17.6%)	9 (52.9%)	5 (29.4%)
3	4 (30.7%)	8 (61.5%)	1 (7.7%)
	*p* = 0.32		
Emboli			
Present	3 (20%)	9 (60%)	3 (20%)
Absent	8 (25%)	14 (29.8%)	10 (21.3%)
	*p* = 0.48		
Histological Grade			
I	3 (25%)	1 (8.3%)	8 (66.7%)
II	4 (28.6%)	9 (64.3%)	1 (7.1%)
III	4 (19%)	13 (61.9%)	4 (19.0)
	*p* = 0.005		

## Data Availability

The raw data supporting the conclusions of this article will be made available by the authors on request.
